# A Simple Step-by-Step Technique for the Management of Gagging in Edentulous Patient

**DOI:** 10.7759/cureus.24423

**Published:** 2022-04-23

**Authors:** Shreya Colvenkar, Varalakshmi Reddy, Suman Thotapalli, Koppunoor Deepa Rani, Sneha Bharadwaj

**Affiliations:** 1 Prosthodontics, MNR Dental College and Hospital, Hyderabad, IND; 2 Prosthodontics, Malla Reddy Dental College for Women, Hyderabad, IND; 3 Prosthodontics, SB Patil Dental College and Hospital, Bidar, IND

**Keywords:** round chocolates, tooth brush, relaxation, training, edentulous, gagging

## Abstract

Gagging presents a clinical challenge to the dentist in all aspects of treatment starting from diagnostic procedures to active treatment. There is no single cause associated with gagging. For successful management, it is very important to find the cause and plan the treatment accordingly. This article describes a simple step-by-step technique for eliminating the gag reflex in an edentulous patient. This simple approach helped the patient to relax and eliminate the phobia of dental treatment. It also enhanced the patient’s ability to continue the dental procedure.

## Introduction

Gagging is one of the common problems encountered by the dentist during clinical practice. Gagging is a normal defense mechanism to prevent the foreign substance from entering the respiratory tract. The patients have extreme sensitivity and they cannot tolerate any foreign material in their mouths. This will be a very stressful experience for the dentist as well as the patient. It can make certain diagnostic procedures to finishing treatment cumbersome as well as time-consuming. Gagging has multifactorial etiology [1,2]. Systemic disorders, anatomic factors as well as psychological disorders can cause patients to gag. Various treatment modalities like behavioral techniques [3], acupressure [4], hypnosis [5], acupuncture [6], systemic desensitization [7], and pharmacological management [8] have been mentioned in the literature. Proper management lies in first finding the cause and treating it. This should be done before the start of the dental procedure. This article describes an easy step-by-step approach to managing a gagging patient.

## Case presentation

A 55-year-old edentulous male patient reported to the department of prosthodontics for replacement of teeth with complete dentures. During the oral examination, the patient experienced a gag reflex on the insertion of the mouth mirror. This made the patient very anxious to carry out the procedure. To successfully manage a gag reflex, the following step-by-step approach was followed.

Patients initial visit

Since the patient presented with gagging on insertion of mouth mirror, no dental procedure was carried out on that day. The patient was attended to with a calm and caring attitude to reduce his anxiety. The patient was involved in an interesting conversation for positive reinforcement. During the conversation, the patient revealed that he had previously started dental treatment and left it halfway because of gagging. The patients gagging was psychogenic in nature. The patient was instructed to carry out two procedures at home, to reduce his anxiety during treatment. He was instructed to stand in front of the mirror and gently massage the posterior area of the palate as far as possible with his toothbrush. He was asked to do this twice, once in the morning and later at night before going to sleep. He was also asked to put four-round chocolates in his mouth one by one at his freedom, till all four chocolates are present in his mouth. He was instructed to do it thrice and not to chew on chocolates. The patient was instructed to follow both procedures for a week.

First appointment

**Figure 1:**
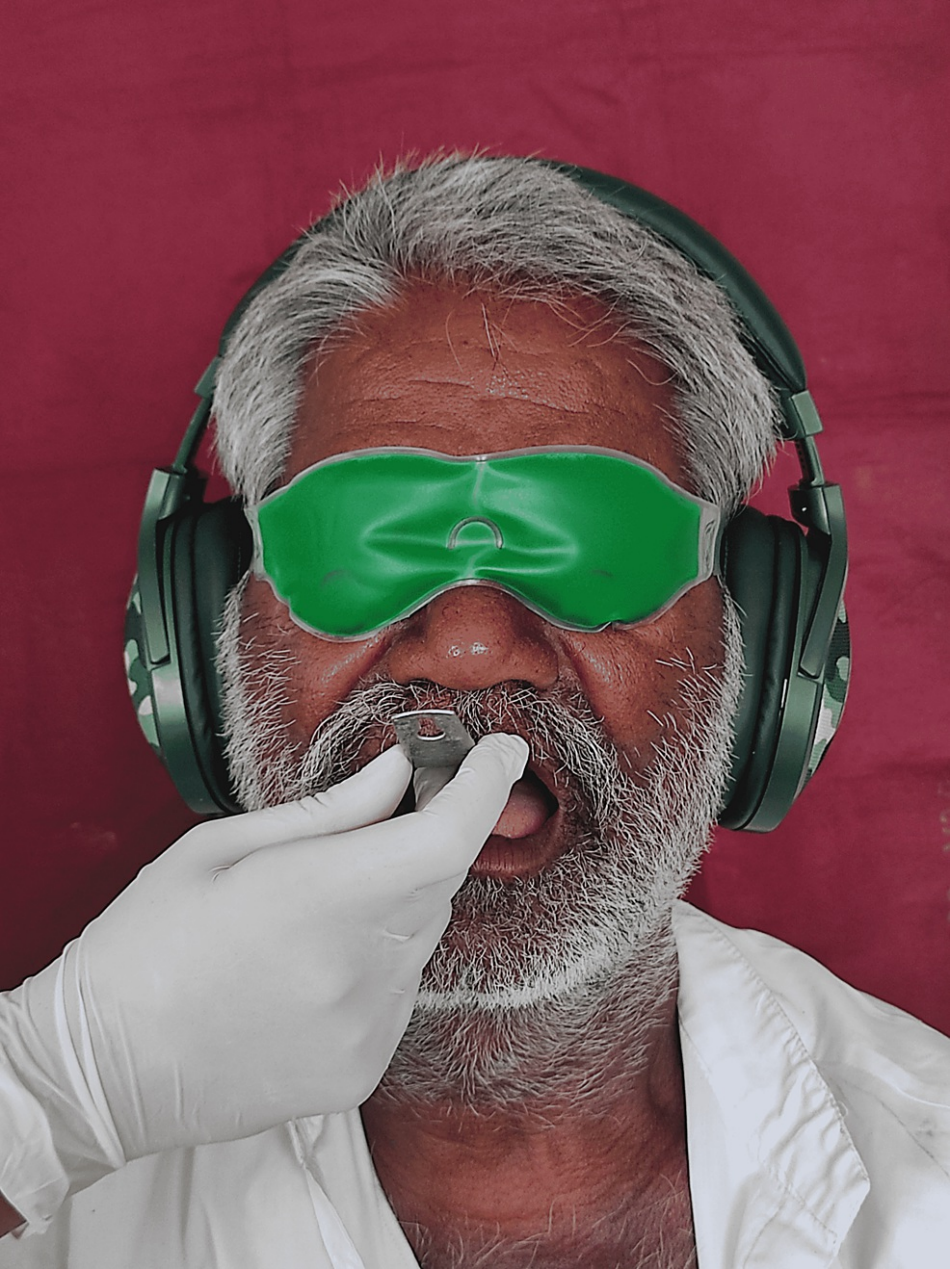
Patient wearing headphones and aloe vera eye mask

Since the patient was able to tolerate chocolates in his mouth, he was assured that he will be able to continue the dental treatment and would be able to wear the dentures. He was also assured that a temporary denture base plate will be given to him so that he gets accustomed to the feel and touch of the final prosthesis. All the steps of denture fabrication were explained to the patient. The distraction technique was followed during impression making. The patient was asked to lift his leg and hold it for some time during impression making. But the distraction technique did not help and the patient had a gagging episode during impression making. The patient was counseled that he will be fine if he tries to conquer his fear. The procedure was stopped only after his consent, it was started again after 30min. At this stage, the patient was asked to wear headphones and listen to meditating music. He was told to practice rhythmic breathing and meditate. The aloe vera eye mask was placed on the eyes and the patient was asked to relax. Once the patient was totally relaxed the procedure was started again. The lower impression tray was inserted into the patient’s mouth. and surprisingly no gagging was experienced by the patient. Impression making was completed at this appointment, with the patient wearing headphones and an aloe vera eye mask (Figure 1).

Second appointment

**Figure 2:**
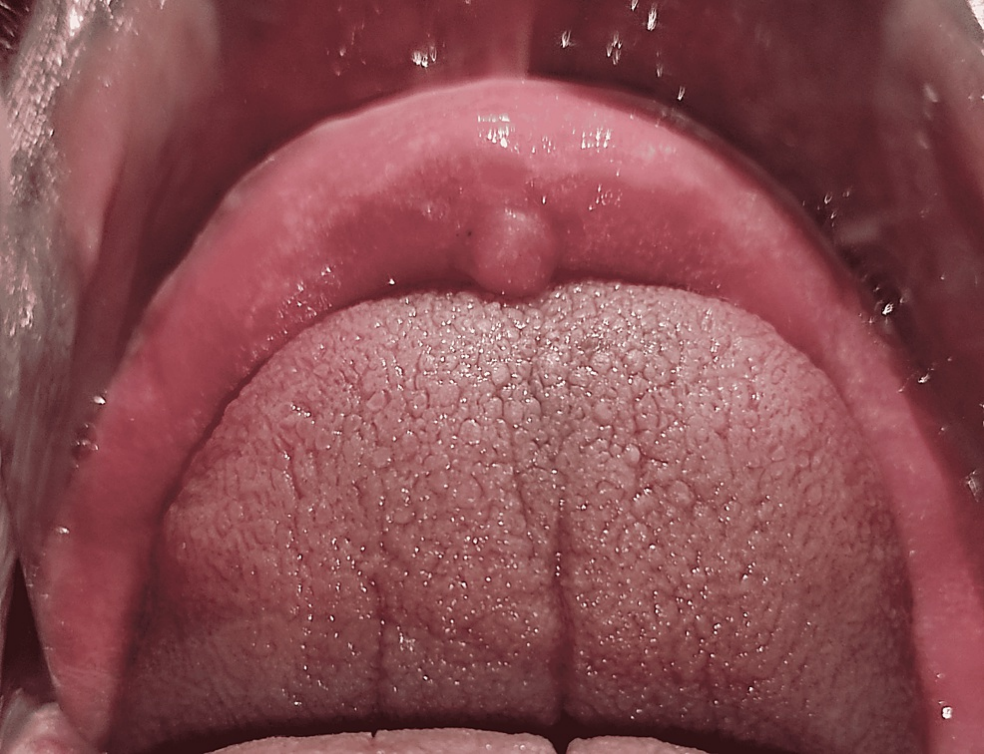
Mandibular training denture base

A mandibular temporary denture base plate with correct extensions was inserted in the patient’s mouth. A small training bead was kept in the anterior region on the lingual side to correctly position the tongue (Figure 2).

The patient was asked to continue the brushing technique together with the chocolate technique till the next visit.

Third appointment

The patient was relaxed again by listening to tranquil music. He was told to meditate and practice rhythmic breathing. This time patients’ eyes were not covered with an aloe vera mask. This would confirm if the techniques he practiced every day, helped him to control gagging. Maxillary impression was made and no gagging was experienced by the patient.

Fourth appointment

**Figure 3:**
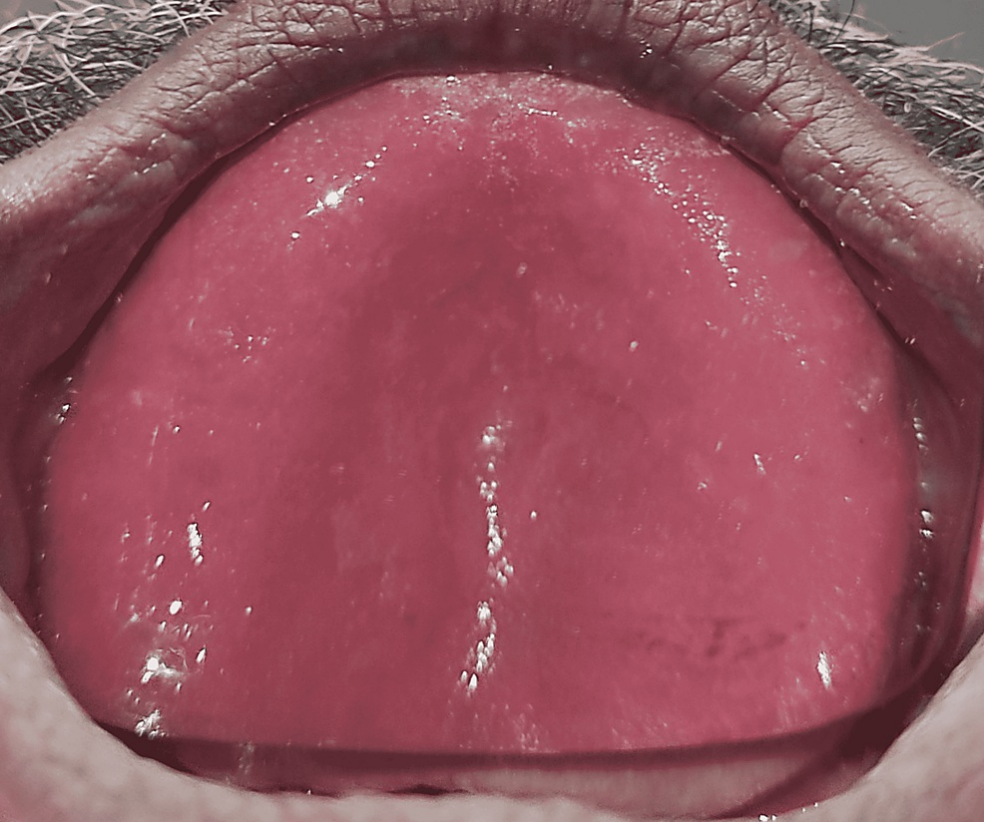
Maxillary training denture base

A maxillary temporary denture base plate was inserted in the patient’s mouth and the patient was asked to continue wearing the trial denture base till the final prosthesis was delivered to the patient (Figure 3).

The patient was instructed to remove it during eating and sleeping. The patient was instructed to wear both the training base plates for a week. The chocolate and toothbrush technique was discontinued.

Remaining appointments

**Figure 4:**
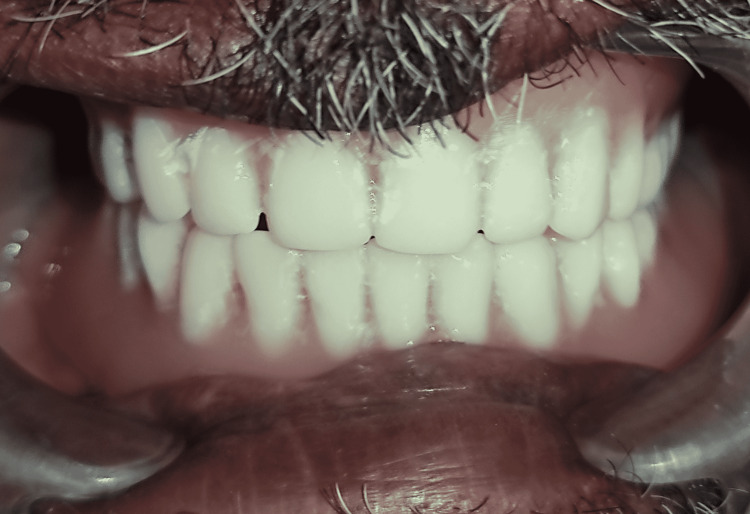
Patient with dentures

**Figure 5:**
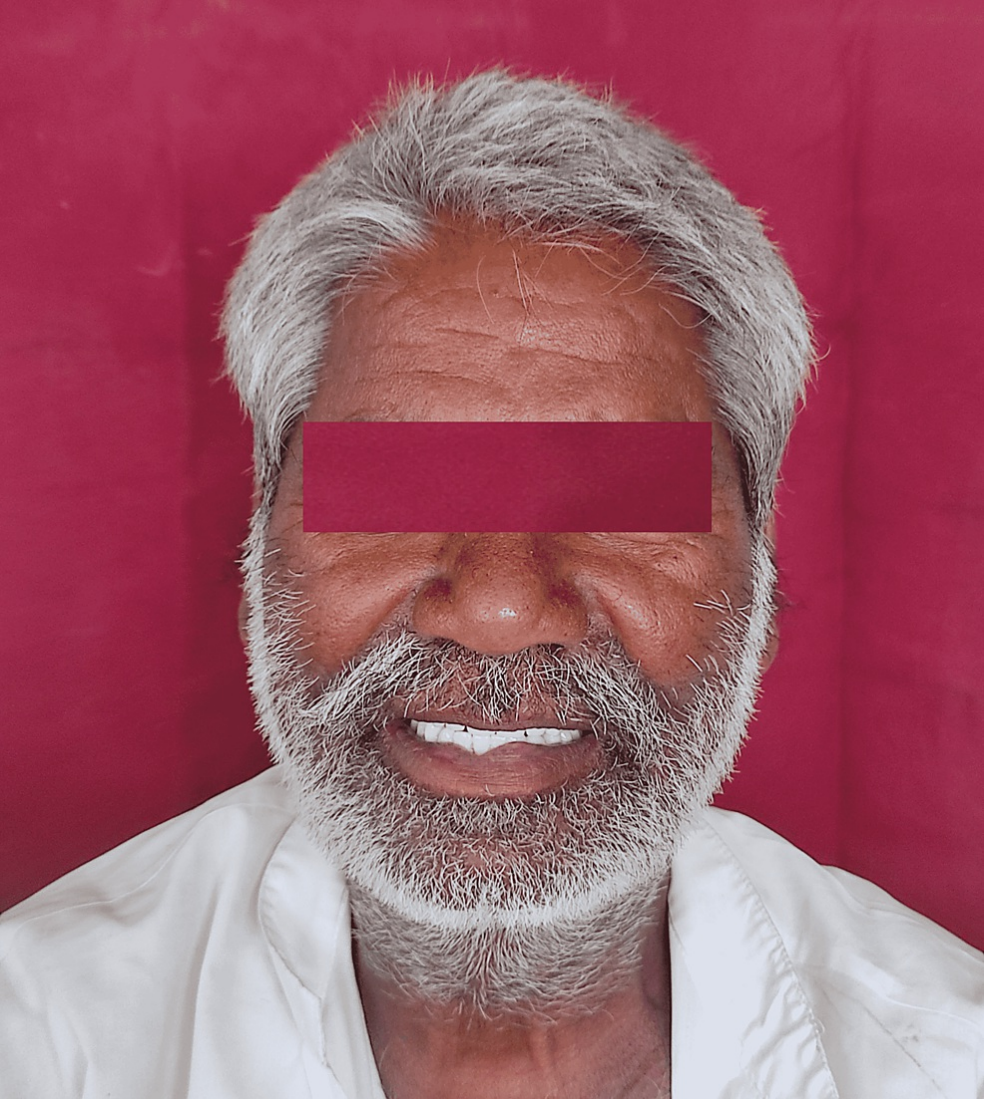
Satisfied denture patient

As the patient was comfortable wearing both the training denture base plate for a week, the procedure for fabrication of a complete denture was started. The remaining steps in complete denture fabrication were completed in subsequent appointments. Complete dentures were successfully delivered to the patient (Figures 4, 5).

## Discussion

The Prosthodontists will frequently encounter patients in the clinics whose oral cavity is extremely sensitive and they cannot tolerate any foreign substance. Gag reflex can be caused by anatomic, medical, or psychosomatic factors. No one cause is associated with it. It is sometimes very difficult to demarcate clearly the etiology of gagging. Hence proper individual assessment of the patient should be carried out to know the exact cause of gagging. This should be completed before starting the treatment for the patient.

In the present case, the patient had anxiety about the treatment. The patient had previously discontinued the treatment because of gagging. It is very important that the patient should be calm and relaxed before the start of the treatment. The empathic and considerate behavior of the clinician will help to alleviate patients’ anxiety. The patient was involved in interesting conversations together with active engagement during treatment. The patient was also given a choice to discontinue the treatment for some time and relax whenever he intended to do so.

The patient was instructed to brush the palate as far as possible with a soft brush twice daily at home. This would habituate the patient to the treatment procedures and finally the denture

The patient was also asked to keep four round chocolate one by one in the mouth for a week. This technique was a modification of the singer’s marble technique [9]. The patient was asked to use chocolates instead of glass marbles due to accidental fear of ingestion. The use of this technique helped the patient to slowly exhaust the gag reflex over time and carry on the procedure without gagging.

The straight left leg technique was used to distract the patient during impression making [10]. But it did not help in any way with gagging. So, the patient was asked to listen to tranquil music using headphones. Also, an aloe vera eye mask was placed on the eyes and the patient was asked to perform meditation with rhythmic breathing. This helped the patient to prevent gag reflex and totally relax him during the procedure. The technique with headphones and aloe vera eye mask has not been used till now.

Prosthodontic management [11,12] to the gagging involves technical modifications like reducing excess thickness and overextension and correcting inadequate post dam to render the prosthesis more acceptable to the patient. The proper selection of impression material is important for gagging patients. The primary impression was made with a high viscosity elastomer material to prevent excessive flow to the gag reflex inducing sensitive areas. The patient was given a training denture bases plate for a week before giving the final prosthesis to achieve better acceptance to complete dentures in the future. A complete denture with a matt finish was given rather than a smooth glossy surface.

It is always good to follow standard techniques than to over-depend on drugs in gagging patients. In the present case, no pharmacological drugs [8] were used to control gaging. Treating the cause rather than symptoms will go a long way in allaying patients' fear of gagging. Patients’ behavior modification in the present case helped to control gagging. This, in turn, depends on the time, patience, and skill of the operator for the success of treatment.

## Conclusions

The gag reflex causes inconvenience to the patient as well as the dentist. If proper diagnosis and treatment planning is not carried out, any treatment is bound to fail. This article describes a simple step-by-step technique to eliminate the gag reflex in an edentulous patient. This simple approach helped the patient to relax and enhanced his ability to continue the dental procedure Though the procedure was time-consuming and required lots of patience, a smile on the patient face after successfully completing the treatment is worth the time and effort.
